# Interactions of an Emerging Fungal Pathogen *Scedosporium aurantiacum* with Human Lung Epithelial Cells

**DOI:** 10.1038/s41598-019-41435-3

**Published:** 2019-03-22

**Authors:** Jashanpreet Kaur, Liisa Kautto, Anahit Penesyan, Wieland Meyer, Liam D. H. Elbourne, Ian T. Paulsen, Helena Nevalainen

**Affiliations:** 1 Department of Molecular Sciences, Macquarie University, Sydney, Australia; 2 Biomolecular Discovery and Design Research Centre, Macquarie University, Sydney, Australia; 3 Molecular Mycology Research Laboratory, Centre for Infectious Diseases and Microbiology, Marie Bashir Institute for Infectious Diseases and Biosecurity, Sydney Medical School - Westmead Hospital, The University of Sydney, Westmead Millennium Institute, Sydney, Australia

## Abstract

*Scedosporium* fungi are found in various natural and host-associated environments, including the lungs of cystic fibrosis patients. However, their role in infection development remains underexplored. Here the attachment of conidia of a virulent *S. aurantiacum* strain WM 06.482 onto the human lung epithelial A549 cells *in vitro* was visualized using microscopy to examine the initial steps of infection. We showed that 75–80% of fungal conidia were bound to the A549 cells within four hours of co-incubation, and started to produce germ tubes. The germinating conidia seemed to invade the cells through the intercellular space, no intracellular uptake of fungal conidia by the airway epithelial cells after conidial attachment. Transcriptomic analysis of the A549 cells revealed that the up-regulated genes were mainly associated with cell repair and inflammatory processes indicating a protective response against *S. aurantiacum* infection. Network analysis of the differentially expressed genes showed activation of the innate immune system (NF-kB pathway) leading to the release of pro-inflammatory cytokines. We believe this is the first report showing the transcriptomic response of human alveolar epithelial cells exposed to *S. aurantiacum* conidia paving a way for better understanding of the mechanism of the infection process.

## Introduction

*Scedosporium* species are ubiquitous fungi, common in the environment. They are also increasingly recognised as colonizers of the lung in cystic fibrosis (CF) and in other forms of chronic lung disease^1–4^. *Scedosporium* spp. have been found at relatively high frequency in environments of high human activity in Australia, Austria and other parts of Europe (reviewed in^5^), which increases the likelihood of acquiring *S. aurantiacum* infection. The small size of *S. aurantiacum* conidia (2–5 µm) can allow them to easily enter the respiratory tract *via* inhalation and traverse to the innermost areas of the lungs^6^. Treatment of *S. aurantiacum* infections is challenging as the fungus is highly resistant to most of the currently used antifungal agents including amphotericin B, 5-flucytosine, the azoles and the echinocandins^7–9^. Despite the above, infections caused by *S. aurantiacum* have been studied to a much lesser extent than those caused by other major lung pathogens such as *Aspergillus fumigatus*.

There is increasing evidence suggesting a key role for the airway epithelium in the response to respiratory pathogens, particularly at early stages of fungal infection (reviewed in^10^). A frequently described mechanism of *in vitro* infection involves attachment of the fungal conidia to the airway epithelial cells and their subsequent internalization. This is followed by degradation of the internalized conidia in the endosomal system and clearing from the host. Some conidia escaping this may germinate and re-enter the extracellular space (reviewed in^10^). It has also been shown that conidia attached onto cell surface produce germ tubes that will aid penetration and thus infection of the host cells^11–14^. Invasion of the host cells and subsequent deployment of defense mechanisms by the host against the pathogen are central to the pathogenesis of the disease^15^.

Infection of cells will evoke a cellular immune response. Cell-mediated immune defense involves cells that can destroy infectious agents through phagocytosis or cytotoxicity. Examples include neutrophils, macrophages, eosinophils, basophils, B- and T-lymphocytes, NK cells (natural killer cells) and cytokines. Another line of host defense towards invasion is through humoral immunity mediated by antibodies and the complement cascade^16^.

Type-II alveolar epithelial cells such as A549 cells have been widely used as a model to study the infection process and host immune response to a large number of CF pathogens including *Pseudomonas aeruginosa*, *Aspergillus fumigatus*, *Candida albicans* and *Cryptococcus neoformans*^17–20^. Interactions between the A549 epithelial cells, viewed as non-professional phagocytes^14^, and *A. fumigatus* have been explored by confocal and scanning electron microscopies^11,12,21^. Recently, proteome and transcriptome based analyses have also gained momentum in identifying molecular mechanisms of the interaction^10,19,22–25^.

Despite the increasing importance of *S. aurantiacum* as an infectious agent, the pathobiology of this opportunistic pathogen is not well known or extensively explored. In this study, we assess interactions between a highly virulent *S. aurantiacum* strain and human airway epithelial cells *in vitro*. The co-culture of A549 cells and *S. aurantiacum* WM 06.482 conidia was visualized using both confocal (CLSM) and scanning electron microscopy (SEM). RNA sequencing was performed to understand the response of the alveolar epithelial cells to the invading pathogen and mechanisms by which the pathogen may trigger the response.

## Results

### Adherence of *S. aurantiacum* conidia to the A549 cells as a function of time

**Figure 1:**
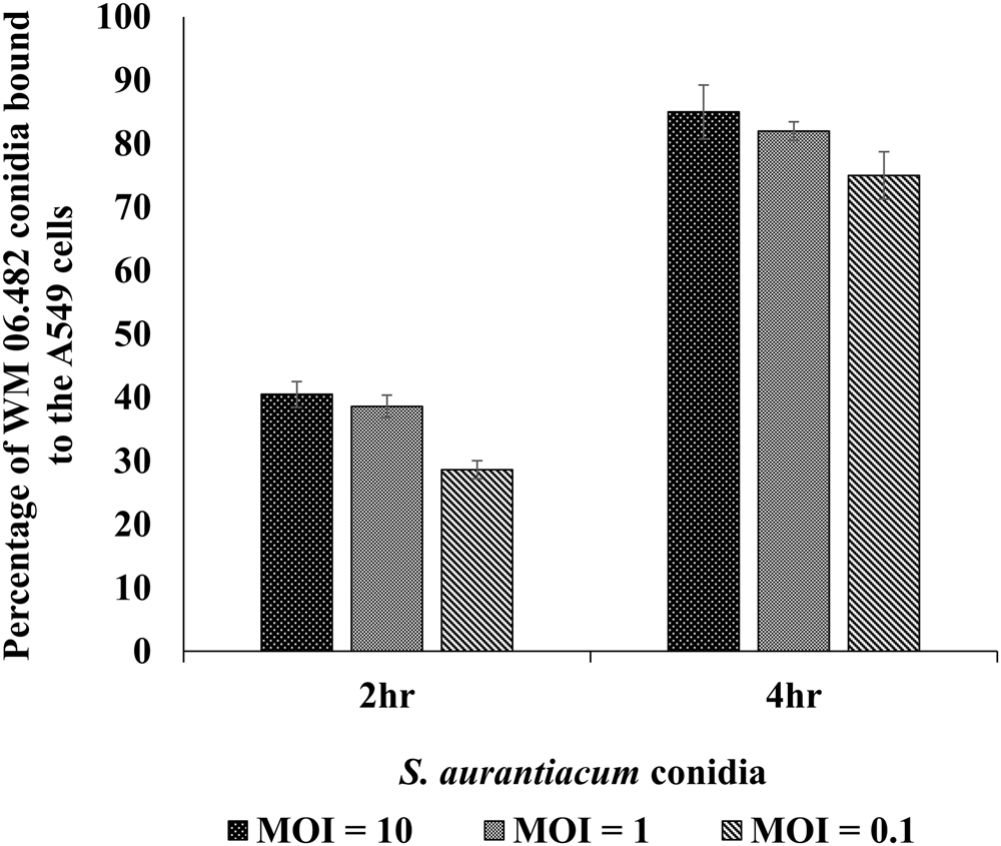
The extent of adhesion of *S. aurantiacum* WM 06.482 conidia to the A549 human lung epithelial cells after 2 h and 4 h, respectively. Error bars represent standard error (±SE) of the mean of three biological replicates for each time point. MOI = multiplicity of infection = number of conidia/cell.

The adherence of *S. aurantiacum* conidia to the airway epithelial cells was determined after co-incubating the A549 cell monolayers with WM 06.482 conidia (MOI = 10, 1 and 0.1 per human cell) at 37 °C for 2 and 4 hours, respectively. The relative extent of adhesion of WM 06.482 conidia to A549 cells was directly proportional to the amount of conidia added at each time point (Fig. 1), with maximum adherence observed for conidia to the A549 cell ratio of 10:1.

About 40% of the fungal conidia were attached to the epithelial cells over a period of 2 h whereas at the end of 4 h co-incubation, the percentage of adhesion increased to about 80% in the A549 cells infected with different doses of WM 06.482 conidia. Therefore, 4 h was chosen for the earliest assessment point for further studies.

### Visualization of co-cultures using CLSM

**Figure 2:**
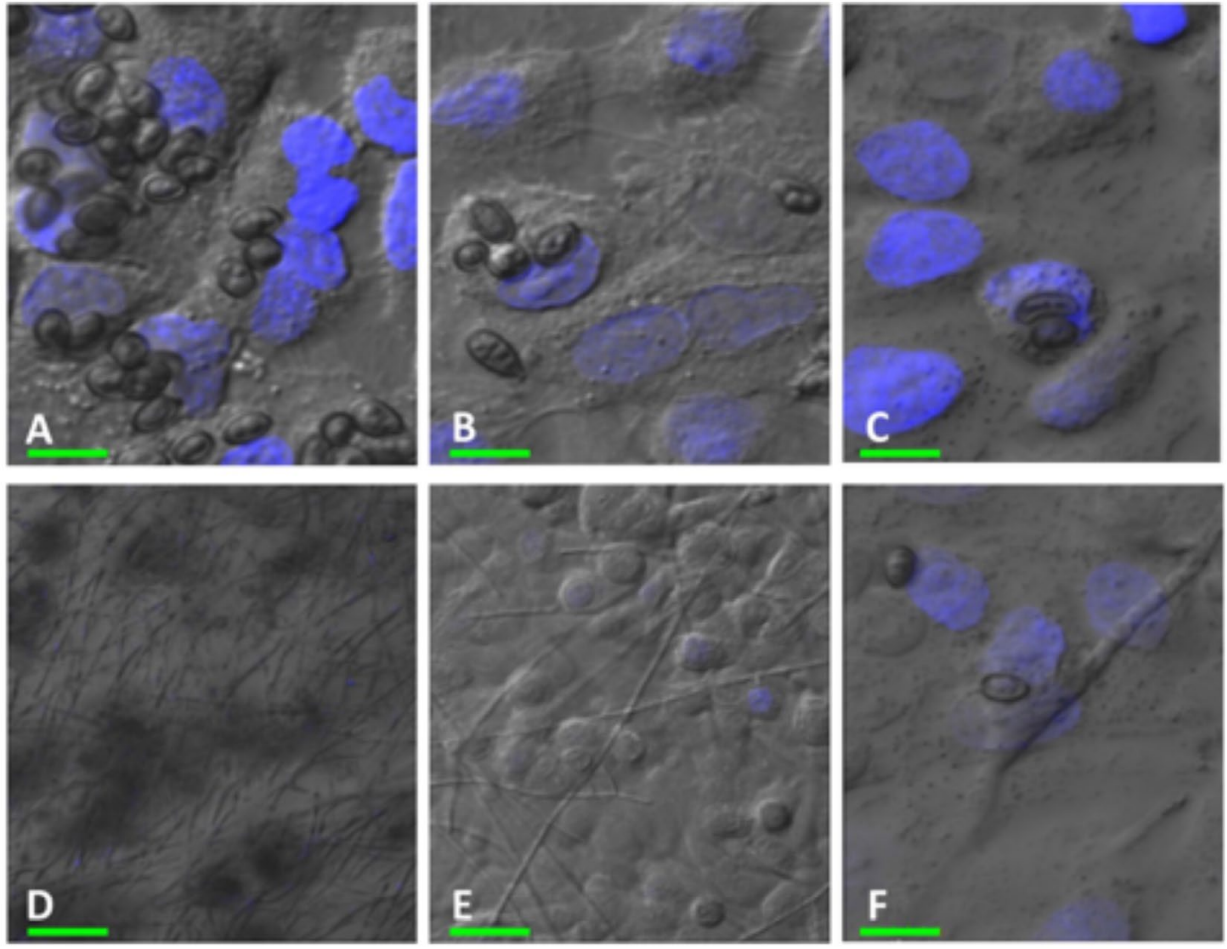
Confocal laser scanning microscopy (CLSM) images of the co-culture of *S. aurantiacum* strain WM 06.482 and A549 cell monolayers after 4 h and 24 h of incubation at 37 °C. The epithelial cells were stained with nucleic acid specific stain Hoechst 33342 (shown in blue). A549 cells after incubation with WM 06.482 conidia (**A**,**D**) MOI = 10:1, (**B**,**E**) MOI = 1:1 and (**C**,**F**) MOI = 0.1:1 for 4 h (**A**–**C**) and 24 h (**D**–**F**), respectively. Scale bars (in green): (**A**) = 5 µm, (**B**,**C** and **F**) = 10 µm and (**D**,**E**) = 20 µm.

The attachment of WM 06.482 conidia to the cultured airway epithelial cells was visualized using confocal microscopy. *S. aurantiacum* conidia attached to the A549 cell monolayers within 4 h of co-incubation as seen in Fig. 2A–C. Fungal attachment to the A549 cells appeared to be specific as no attachment was observed on the glass coverslips without the epithelial cells.

CLSM images after 4 h (Fig. 2A–C) of co-incubation showed that human lung epithelial cells could carry more than one *S. aurantiacum* conidium and the conidia were mainly attached to the cell surface. Following 24 h of co-incubation (Fig. 2D,F), most of the fungal conidia had germinated and formed a network of hyphae on the A549 cells. Conidia to A549 cell ratio of 1:1 was considered optimal for the binding of WM 06.482 conidia to the A549 cells (Fig. 2B,E) as a higher fungal burden (10:1, Fig. 2A,D) resulted in greater cell damage, and a lower fungal load (0.1:1, Fig. 2C,F) was not sufficient to cause an effect.

### Interaction of *S. aurantiacum* conidia with A549 cells visualized by SEM

Interaction between *S. aurantiacum* WM 06.482 and human lung epithelial cells were further characterized using SEM. The cultures were constituted with a conidia to A549 cell ratio of 1:1 and maintained for 4, 8 and 24 h respectively to assess the interactions at different stages of conidial germination.

**Figure 3:**
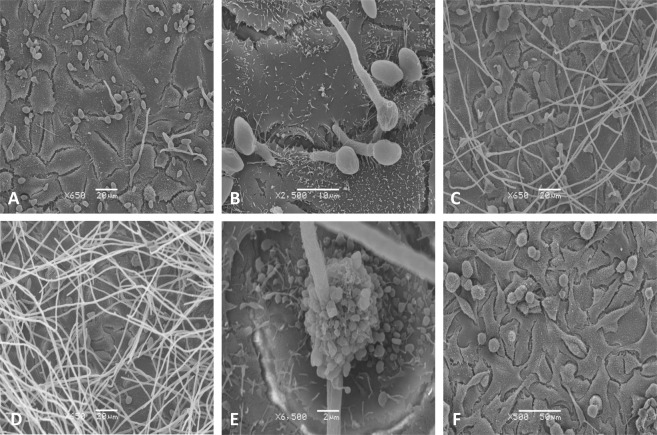
The invasion of A549 lung epithelial cells by *S. aurantiacum* WM 06.482 observed by scanning electron microscopy (SEM). (**A**) WM 06.482 conidia adhered to A549 cells after 4 h of incubation (x 650). (**B**) Germ tube projections from the WM 06.482 conidia attached to the A549 interstitial sites (x 2500). (**C**) A549 cells covered with WM 06.482 hyphae after 8 h of co-incubation (x 650). (**D**) WM 06.482 hyphae completely covering the A549 cells after 24 h (x 650). (**E**) Rounding of A549 cells after 8 h (x 6500). (**F**) Conidia free A549 control cells maintained for 24 h (x 500). Scale bars (**A**,**C** and **D**) = 20 µm, (**B**) = 10 µm, (**E**) = 2 µm and (**F**) = 50 µm.

SEM analysis of co-cultures showed that after 4 h of incubation most of the *S. aurantiacum* conidia were attached to the A549 cell surface in small groups (Fig. 3A). Small germ tube projections were observed that were mainly present in the interstitial regions between the human lung epithelial cells indicating penetration of the epithelial cell membrane with the help of the germ tubes (Fig. 3B). At a later time point, at 8 h, germinating WM 06.482 conidia had penetrated numerous A549 cells (Fig. 3C), while some human lung epithelial cells still appeared morphologically intact. By the end of 24 h (Fig. 3D), the A549 cells were completely covered by *S. aurantiacum* hyphae and damaged cells showed distinct signs of membrane blebbing and apoptosis visualized by the appearance of a large number of small sphere-like apoptotic bodies (Fig. 3E). After 24 h, the A549 cells were completely detached from the culture plate whereas conidia-free control cells remained adhered (Fig. 3F). There were no signs of internalization of the conidia into the A549 cells at any time point studied.

### Transcriptome analysis of A549 human epithelial cells response to the *S. aurantiacum* infection

RNA sequencing was carried out for A549 lung epithelial cells infected with live WM 06.482 conidia in order to investigate gene expression changes in the airway epithelial cells in response to *S. aurantiacum* infection. RNA was extracted from the A549 cells after 8 h of exposion to WM 06.482 and from non-infected A549 cells maintained for the same amount of time, and sequenced. Analysis of the RNA sequencing data revealed ~3950 differentially expressed genes (*q val < *0.05) in A549 cells infected with WM 06.482 compared to uninfected cells, which comprises nearly 20% of all predicted gene sequences. Out of this statistically significant list of genes, 2008 genes were found to be up-regulated and 1942 were down-regulated. The complete list of genes is provided as a supplementary file (Supplementary Table S1). Many of the highly differentially expressed genes were linked to the cell mediated immune defense and are discussed further below.

### Functional annotation of the differentially expressed genes

Functional annotation and gene ontology (GO) classification of genes listed in Supplementary Table S1 was performed using the DAVID online tool (http://david.abcc.ncifcrf.gov/). The GO categories significantly over represented among the up-regulated and down-regulated genes are shown in Table 1(A) and (B) respectively. The up-regulated genes showed enrichment of GO terms associated with cell death, inflammation and signalling molecules such as cytokines, whereas GO terms categories for down-regulated genes included processes related to cell cycle progression such as cell division, mitosis and chromosome segregation.

**Table 1:** Gene ontology (GO) term annotation for the list of differentially expressed genes. (A) List of gene ontology terms associated with up-regulated genes. (B) List of gene ontology terms associated with down-regulated genes. BP = biological process, MF = molecular function and CC = cellular component. P-values represent the significance of functional association as calculated by DAVID.

GO ID	Function	Ontology	p-value
**A. Upregulated**			
GO:0009611	Response to wounding	BP	1.63 × 10e-7
GO:0008219	Cell death	BP	8.09 × 10e-6
GO:0007155	Cell adhesion	BP	6.41 × 10e-6
GO:0006955	Immune response	BP	1.07 × 10e-6
GO:0006954	Inflammatory response	BP	5.30 × 10e-5
GO:0005125	Cytokine activity	MF	2.32 × 10e-5
GO:0009991	Response to external stimulus	BP	1.01 × 10e-4
GO:0030036	Actin cytoskeleton organisation	BP	7.34 × 10e-4
GO:0006915	Apoptosis	BP	9.48 × 10e-4
**B. Downregulated**			
GO:0000279	M-phase	BP	9.82 × 10e-7
GO:0022402	Cell cycle process	BP	7.63 × 10e-7
GO:0051301	Cell division	BP	1.05 × 10e-6
GO:0000278	Mitotic cell cycle	BP	1.63 × 10e-6
GO:0007067	Mitosis	BP	1.17 × 10e-6
GO:0048285	Organelle fission	BP	7.85 × 10e-5
GO:0007098	Centrosome cycle	BP	1.43 × 10e-5
GO:0043229	Intracellular organelle	CC	4.40 × 10e-4
GO:0007059	Chromosome segregation	CC	1.74 × 10e-3
GO:0000280	Nuclear division	BP	1.17 × 10e-2

### Pathway detection and gene network analysis

**Figure 4:**
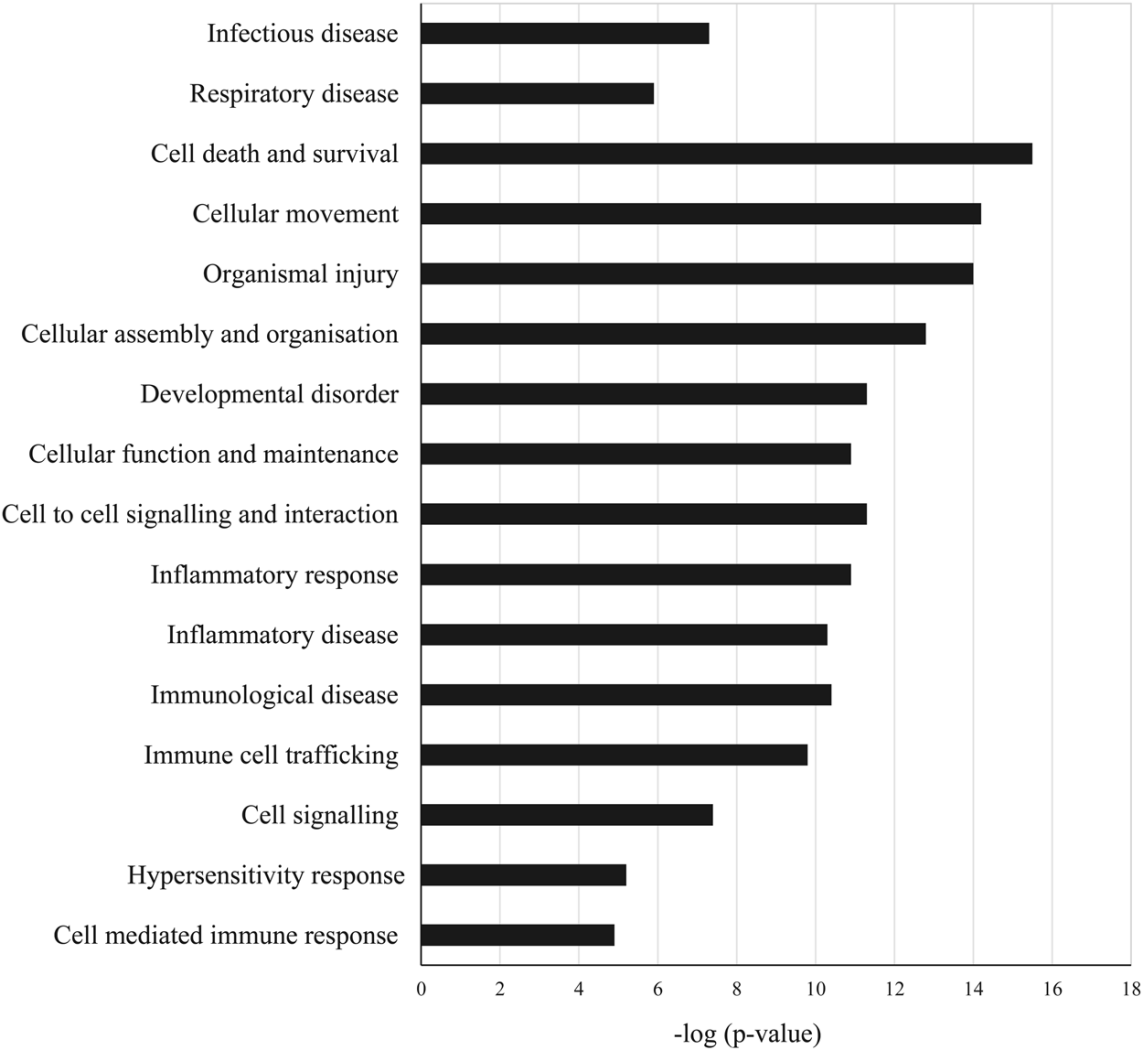
Biological processes in the A549 human lung epithelial cells affected by the infection with *S. aurantiacum* WM 06.482 conidia. Y-axis represents the biological processes and x-axis is the −log (p-value) as obtained after IPA analysis.

Significantly differentially expressed genes (listed in Supplementary Table S1) were further analysed using Ingenuity Pathway Analysis tool (IPA, http://www.ingenuity.com) for the detection of biological pathways and gene networks (Supplementary Fig. S1) in A 549 cells in response to fungal infection. Biological processes for the corresponding gene sets were mainly related to cell death and survival, organismal injury, and inflammatory and immune responses (Fig. 4).

Based on the information contained in Table 1(A), Fig. 4 and Supplementary Fig. S1, inflammation plays an important role in the response of A549 cells to WM 06.482. The pathway map for inflammation revealed interactions between several upregulated genes that pertain to NF-kB (nuclear factor kappa-light-chain-enhancer of activated B cells) complex and/or NF-kB family such as *NFKB2* and *RELB*, along with genes encoding transcription regulators NFKBID (inhibitor, delta), JUNB and ATF3 (activating transcription factor 3). Genes coding for interleukins such as IL11 and CXCL8 (interleukin 8), were also upregulated. The only gene that was downregulated in the network was *TXNIP* (thioredoxin-interacting protein) (Supplementary Table 1).

**Figure 5:**
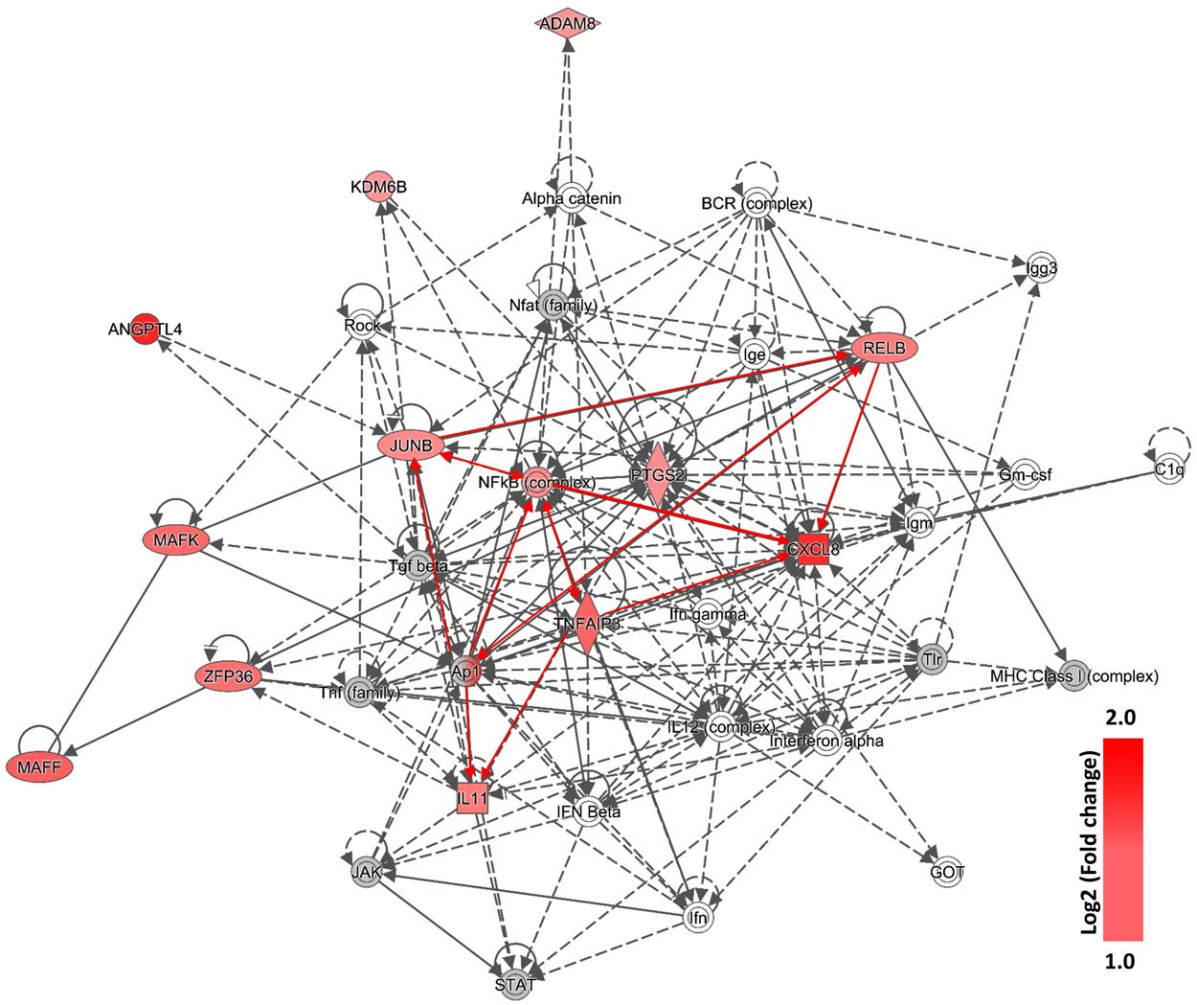
Sub-network of the innate signaling pathway obtained after IPA analysis of the differentially expressed genes in A549 lung epithelial cells infected with WM 06.482 conidia. Significantly up-regulated genes are shown in red, the intensity of red color shows the level of up-regulation of the gene expressed as log2 (fold change). The genes that did not show any changes in expression after 8 h of incubation with WM 06.482 are shown in grey. The full network is presented as Supplementary Fig. S1.

Further analysis of the subnetwork of immunological cluster (Fig. 5) revealed that integrated signaling between transcription factors such as JUNB and TNFAIP3, and members of NF-kB family including *RELB* and *NFKB2*, might be involved in the up-regulation of pro-inflammatory cytokines such as IL11 and CXCL8.

### Validation of selected differentially expressed genes using qRT-PCR

Five genes involved in the immune response, i.e. *RELB*, *ATF3*, *TNFAIP3*, *MUC5B* and *CXCL8*, were selected to validate the transcriptomic data by qRT-PCR. *CXCL8* showed highest up-regulation (8 fold) in the A549 cells infected with *S. aurantiacum* compared to the A549 cells alone (Supplementary Fig. S2). *ATF3*, *TNFAIP3* and *MUC5B* were up-regulated 5.34 fold, 4.2 fold and 4.6 fold respectively. A low level but significant up-regulation was also observed for *RELB* (1.77 fold). No amplification was observed for RNA samples, which confirmed the purity of cDNA.

## Discussion

There are published reports on the production of inflammatory mediators by the human lung epithelial cell line A549 after stimulation with fungi or fungal extracts^26–28^, and after internalization of fungal conidia (reviewed in^10^ and^14^). In contrast, very little information exists on the response of the A549 cells to conidia germinating on the cell surface. To the best of our knowledge, interaction of *S. aurantiacum* with the A549 cells has not been explored in any detail previously. There is a recent microscopy study on the interaction of *S. apiospermum* with A549 lung epithelial cells^29^, which, however, does not include transcriptomic/network analyses of interactions which are presented in this study.

The CLSM and SEM images (Figs 2 and 3) obtained in our study showed attachment of conidia of the highly virulent clinical isolate *S. aurantiacum* WM 06 on the surface of the A549 airway epithelial cells with no signs of cellular internalization during the observation period (4–24 h). This seems different to a number of previous studies with *A. fumigatus* that have shown rapid intracellular uptake of fungal conidia within the airway epithelial cells after an initial adhesion step^19,30,31^.

Electron microscopy studies on *S. aurantiacum* further demonstrated that attachment of conidia to the A549 human lung epithelial cells was accompanied by rapid formation of germ tubes. The appearance of germ tubes as early as 4 h into co-cultivation suggests an active participation of the fungus in the interaction process. Furthermore, the SEM images (Fig. 3B) showed that germ tubes were able to penetrate the epithelial cells through the intercellular spaces with almost all cells having been invaded by the fungal hyphae within 24 h. Consequently, the infected cells lost membrane integrity demonstrating membrane blebbing (Fig. 3E) whereas morphological integrity of the few non-infected cells appeared preserved. Membrane blebbing is considered a sign of the onset of apoptosis in cells as a response to stress factors and allergens^32^. Fungal growth, rather than ingestion of conidia appeared to be the main stimulus for the production of inflammatory mediators by epithelial cells in the studies with *A. fumigatus*^13^.

While the immune reaction of the human lung epithelial cells against *S. aurantiacum* can be deciphered at the gene expression level, the mechanism by which the fungal invader penetrates the epithelial cell layer is not accessible as yet. The restricting factor for the identification and analysis of specific genes and cellular mechanisms involved in pathobiology of this organism is the current limited genomic information on *S. aurantiacum*^33^. In transcriptional profiling of the response of the A549 airway epithelial cells to germinating WM 06.482 conidia at 8 h into co-culture, 3950 genes were found to be differentially expressed in the infected cells compared to non-infected A549 cells maintained for the same length of time (8 h). In particular, the cells showed increased levels of transcripts from genes associated with wound healing, cell repair and inflammatory processes (*e.g*. chemokines) (Table 1A and Supplementary Table S1). *MUC5* involved in mucin production was also found to be up-regulated which indicates that the cells might initiate a mucociliary clearance response against *S. aurantiacum* conidia. Down-regulated genes were mainly involved in cell cycle progression, which suggests a reduction in cell proliferation in response to fungal infection (Table 1B and Supplementary Table S1). Reduction in cell cycle progression has also been reported for human lung epithelial cells infected with *A. fumigatus*^19^.

*S. aurantiacum* conidia germinating on the cell surface led to upregulation of genes for actin cytoskeleton organization (Table 1A). This may be a consequence of a loss of focal adhesions and depolymerization of the F-actin cytoskeleton similarly to that observed with *A. fumigatus*^14^. The responsive pathways activated in the A549 cells by *S. aurantiacum* conidia producing germ tubes on the cell surface seem, overall, to be rather similar to those caused by internalization of *A. fumigatus* conidia^19,25^ although direct comparisons are difficult due to the complexity of the interactions. However, differently to *Aspergillus*, genes and pathways related to phagocytosis or production of proteases, seen in a number of *A. fumigatus* infections (reviewed in^19^), were not amongst the most highly upregulated events in A549 cells infected by *S. aurantiacum*. This may reflect the observation that the fungus seems to invade the cells through interstitial cell spaces and not by cellular intake of conidia. Infection through interstitial cell spaces is clearly demonstrated in Fig. 3(B).

Network analysis of the differentially expressed genes revealed up-regulation of the inflammation pathway in the A549 cells in response to infection with *S. aurantiacum* (Fig. 5). Genes that showed the highest degree of up-regulation in the inflammation pathway were those encoding the two chemokines CXCL8/IL8 and IL11 and members of NF-kB family involving *RELB* and their transcriptional regulators such as *TNFAIP3* and *ATF3*. Previous studies have reported an NF-kB mediated increase in the level of IL8 production in the human respiratory epithelial cells exposed to other fungi including *A. fumigatus* and *Alternaria alternata*, and bacteria such as *P. aeruginosa*^13,26,34–38^. Our dataset also revealed the presence of ADAM8 metalloprotease, which is known to protect the lungs against allergic pulmonary disease, and can be induced by allergens and Th2 cytokines^39^. Together these findings suggest that human alveolar cells could recognize the germinating *S. aurantiacum* conidia on the cell surface and initiate a host defense response by secreting inflammatory cytokines *via* NF-kB pathway.

To the best of our knowledge, this is the first study describing the transcriptomic response of human alveolar epithelial cells exposed to *S. aurantiacum* conidia. This study helps to understand interactions between *S. aurantiacum* and human lung epithelial cells and paves the way for future studies aimed at assessing the detailed mechanisms of hyphal invasion of the human airway epithelial cells.

## Methods

### Organisms and growth conditions

A highly virulent *S. aurantiacum* WM 06.482 (CBS 136046) strain^40^ was obtained from the culture collection of the Medical Mycology Research Laboratory, Centre for Infectious Diseases and Microbiology, Westmead Hospital, Sydney, Australia. Conidia were prepared and stored as described previously^40,41^.

The A549 human epithelial cell line derived from a lung carcinoma was obtained from the American Type Culture Collection (ATCC^®^ CCL-185^™^). The A549 cells were maintained in RPMI 1640 medium (Life technologies, Australia) supplemented with 10% (v/v) FBS (fetal bovine serum, Life technologies, Australia), 1 mM glutamine (Life technologies, Australia), 100 U/ml penicillin and 100 µg/ml streptomycin in cell culture flasks (Sigma-Aldrich, Australia) at 37 °C in a humidified 5% CO_2_ atmosphere. Cell count and viability were calculated with TC20^TM^ Automated Cell Counter (Biorad, Australia) according to the manufacturer’s protocol. Cells were then seeded in RPMI 1640 medium in 8-chamber tissue culture slides (BD Falcon™ CultureSlides) approximately 1 × 10^5^ cells/well at 37 °C.

### Measurement of adherence of *S. aurantiacum* conidia to airway epithelial cells

The confluent A549 cell monolayers seeded in 8-chamber tissue culture slides were incubated with 0.1:1, 1:1 and 10:1 multiplicity of infection (MOI = number of conidia/cell) of *S. aurantiacum* conidia in 500 µl of RPMI 1640 medium for 2 h and 4 h respectively at 37 °C. The co-culture of epithelial cells with each MOI of *S. aurantiacum* conidia was maintained in three independent biological replicates. After incubation, unbound conidia were removed by washing three times with 1 ml of Phosphate Buffered Saline, pH 7.4 (PBS, P4417, Sigma-Aldrich) and epithelial cell monolayers were detached from the plate using trypsin (as described earlier). *S. aurantiacum* conidia were detached using 400 µl of 0.5% (v/v) Triton X-100 and serial dilutions (10-fold) of the released conidia were plated on solid potato dextrose medium (CM0139, Thermo Fisher Scientific) three replicates/well) for 24 h at 37 °C to determine the number of adhered conidia per well.

### Confocal laser scanning microscopy (CLSM)

The co-cultures of *S. aurantiacum* (MOI = 1 conidia/cell) and A549 cells (1 × 10^5^ cells/well) were performed in triplicate in Falcon 8-chamber tissue culture slides at 37 °C for 4 h and 24 h respectively in a humidified 5% CO_2_ atmosphere. After indicated time intervals, the co-cultures were washed with 1 ml PBS to remove unbound *S. aurantiacum* conidia and fixed with 2% (v/v) paraformaldehyde (Sigma-Aldrich) in PBS for 1 h at room temperature. Hoechst 33342 (1:100 of 10 mg/ml stock in PBS, Life Technologies) was used to stain cell nuclei for 15 minutes at room temperature. Images were acquired using a Fluoview FV1000 IX81 Inverted Confocal Microscope (Olympus) with an excitation and emission wavelength of 350/461 nm. Three-dimensional reconstructions of the cell monolayers and associated conidia were also acquired by taking a series of images in the Z-plane. At least 10 microscopy fields were studied per each MOI, representative images are shown.

### Scanning Electron Microscopy (SEM)

The co-cultures were prepared as above and an additional time point of 8 h was included to assess the interactions during germination of fungal conidia. At the end of each incubation period, the co-cultures were washed with PBS and fixed in 3% (v/v) glutaraldehyde (ProSciTech, Australia) in PBS. The samples were dehydrated using a series of ethanol concentrations (30–100%) and dried to the critical point using a K850 critical point dryer (Emitech). Dried specimens were mounted on the specimen stubs and coated with gold particles using K550 gold splutter coater unit (Emitech) and visualized using JSM-7100F Field Emission Scanning Electron Microscope (Jeol) at 10 kV working voltage. At least 10 microscopy fields were studied per each sample, representative images are shown.

### RNA extraction

Confluent monolayers of A549 cells were cultured in triplicate in the Greiner CELLSTAR^R^ dishes (Sigma-Aldrich, Australia) with and without *S. aurantiacum* conidia (MOI = 1 conidium/cell) for 8 h at 37 °C. Upon completion of the incubation, the co-cultures were detached from the culture dish using trypsin (as described previously) and centrifuged for 5 min at 1000 rpm and 4 °C to harvest the cells. The cells were then washed three times with PBS to remove traces of medium and stored at −80 °C. RNA extraction was performed using the TRizol method adopted from^42^. The RNA pellet was purified using RNeasy MinElute Cleanup kit (Qiagen) according to manufacturer’s instructions, dried and resuspended in 20 µl of DEPC-treated water (diethyl-pyrocarbonate, Sigma-Aldrich). RNA concentration was measured using a NanoDrop Spectrophotometer. The quality of RNA was assessed by running 1–1.5 µg of the RNA samples on 1% w/v agarose gel stained with GelRed (Biotium, Australia). The RNA samples were sequenced at the ACRF Biomolecular Resource Facility, John Curtin School of Medical Research, Canberra, Australia.

### Transcriptome analysis

The forward and reverse RNA-Seq Illumina paired-end sequence reads for A549 cells infected by WM 06.482 and non-infected control A549 RNA samples (in biological triplicates) were trimmed to remove adapter sequences and low-quality sequences using Trimmomatic tool^43^. Trimmed high-quality sequence reads were mapped against human genome (*Homo sapiens*, UCSC release hg19) using Tophat2 software^44^. Transcript assembly was performed with Cufflinks software tool; differential expression levels and statistical significance for each gene were calculated with Cuffdiff^45^. Differential expression of key genes was confirmed by a quantitative reverse transcriptase polymerase chain reaction (qRT-PCR, see below).

### Functional annotation of differentially expressed genes and pathway analysis

Statistically significant (*q val*. < 0.05) differentially expressed genes were analyzed using DAVID (The Database for Annotation, Visualization and Integrated Discovery) functional annotation tool, version 6.7 (http://david.abcc.ncifcrf.gov/). The gene dataset was uploaded to DAVID in a tab delimited format and mapped against the *Homo sapiens* reference database to extract information including gene ontology terms, molecular function, biological processes and important pathways. The DAVID functional analysis tool was used with a threshold count of 1.0 and EASE threshold of 0.5. Network analysis for the corresponding gene sets was performed using IPA (Ingenuity Pathway Analysis), version 21249400, released in March 2015 (www.qiagen.com/ingenuity). The list of significantly differentially expressed genes was uploaded to the IPA software and mapped against a reference knowledge base that contains curated information from the published scientific literature. Genes that successfully mapped to the knowledge base were then used to generate the biological pathways and their associated networks.

### Quantitative (q) RT-PCR

Quantitative reverse transcriptase polymerase chain reaction (qRT-PCR) was performed on a selection of differentially expressed genes to validate the results from the transcriptome analysis. Total RNA extracted from A549 cells cultured with and without *S. aurantiacum* conidia was subjected to DNase digestion using DNA-free Turbo DNase Digestion Kit (Ambion) and reverse transcribed to cDNA using QuantiTect® Reverse Transcription Kit (Qiagen) following the manufacturer’s instructions. A standard 10 µl real time-PCR reaction mix comprised of 2 µl of cDNA (2.5 ng/µl) template, 3 µl forward and reverse primers (2 µM each) and 5 µl of Superscript III Platinum SYBR Green One master mix (Invitrogen). All reactions were carried in triplicate in a qTOWER 2.2 (Analytik, Jena) with an initial denaturation of 95 °C for 10 min followed by 40 cycles of 95 °C for 30 sec, annealing at 55 °C for 30 sec and extension at 72 °C for 30 sec.

The primers for each gene (Supplementary Table S2) were designed using Primer3^46^ and assessed for their amplification efficiencies. PCR reactions were also set for RNA only samples to check gDNA contamination in the RNA samples and confirm the quality of cDNA. Housekeeping gene GAPDH was included as an internal reference. Cycle threshold (C_T_) values were exported from the qPCRsoft 2.0 software and the relative abundance of RNA was calculated in each sample using ΔΔC_T_ method^47^.

## Supplementary information

Supplementary dataset 1

Supplementary table S1

## Data Availability

RNA sequence data from this study have been deposited in NCBI Sequence Read Archive (SRA) with the SRA accession: PRJNA495879.
