# Supplementary material for: Interactions of an Emerging Fungal Pathogen Scedosporium aurantiacum with Human Lung Epithelial Cells
Source: Sci Rep. 2019 Mar 22;9:5035. doi: 10.1038/s41598-019-41435-3 (PMC6430781; doi:10.1038/s41598-019-41435-3)

**Interactions of an Emerging Fungal Pathogen *Scedosporium aurantiacum* with Human Lung Epithelial Cells**

Jashanpreet Kaur^1,2^, Liisa Kautto^1,2^, Anahit Penesyan^1,2^, Wieland Meyer^3^, Liam D. H. Elbourne^1,2^, Ian T. Paulsen^1,2^, Helena Nevalainen^1,2*^

^1^Department of Molecular Sciences, Macquarie University, Sydney, Australia

^2^Biomolecular Discovery and Design Research Centre, Macquarie University, Australia

^3^Molecular Mycology Research Laboratory, Centre for Infectious Diseases and Microbiology, Marie Bashir Institute for Infectious Diseases and Biosecurity, Sydney Medical School - Westmead Hospital, The University of Sydney, Westmead Millennium Institute, Sydney, Australia

*Corresponding author: E-mail: [helena.nevalainen@mq.edu.au](mailto:helena.nevalainen@mq.edu.au)

**Supplementary Table S1**. Differentially expressed genes in human lung epithelial cells exposed to *S. aurantiacum* strain WM 06.482 (attached as a separate excel file).

**Supplementary Table S2**. Primer sequences used for qRT-PCR validation of selected differentially expressed genes.

| **Gene** | **Forward (5’-3’)** | **Reverse (5’-3’)** |
| --- | --- | --- |
| GAPDH | CTCTGACTTCAACAGCGAC | TGGTCCAGGGGTCTTACT |
| CXCL8 | TCCTGATTTCTGCAGCTCTG | GTCCACTCTCAATCACTCTCAG |
| ATF3 | CCTCGGAAGTGAGTGCTTCT | ATGGCAAACCTCAGCTCTTC |
| MUC5B | CACATCCACCCTTCCAAC | GGCTCATTGTCGTCTCTG |
| TNFAIP3 | AAAGCCCCTCATCGACAGAAA | CAGTTGCCAGCGGAATTTA |
| RELB | CATCCTGGACCACTTCCTGCC | GAACATGTTGCTGCCCACAAG |

**Supplementary Figure S1.** Innate signalling pathway obtained after IPA analysis of the genes that were differentially expressed in the A549 human lung epithelial cells infected with *S. aurantiacum* WM 06.482 conidia. Red and green colors in the network map symbolize significantly up and down regulated genes, respectively. The intensity of the red colour indicates the level of up-regulation of the gene expressed as log2 (fold change). Grey colour indicates genes that did not show any changes in expression after 8 h of incubation with WM 06.482.

**Supplementary Figure S2.** Relative expression of selected genes in the A549 human lung epithelial cells exposed to *S. aurantiacum* WM 06.482 conidia for 8 h, as assessed by the qRT-PCR. Error bars represent standard error (±SE) of the mean of three biological replicates for each time point.


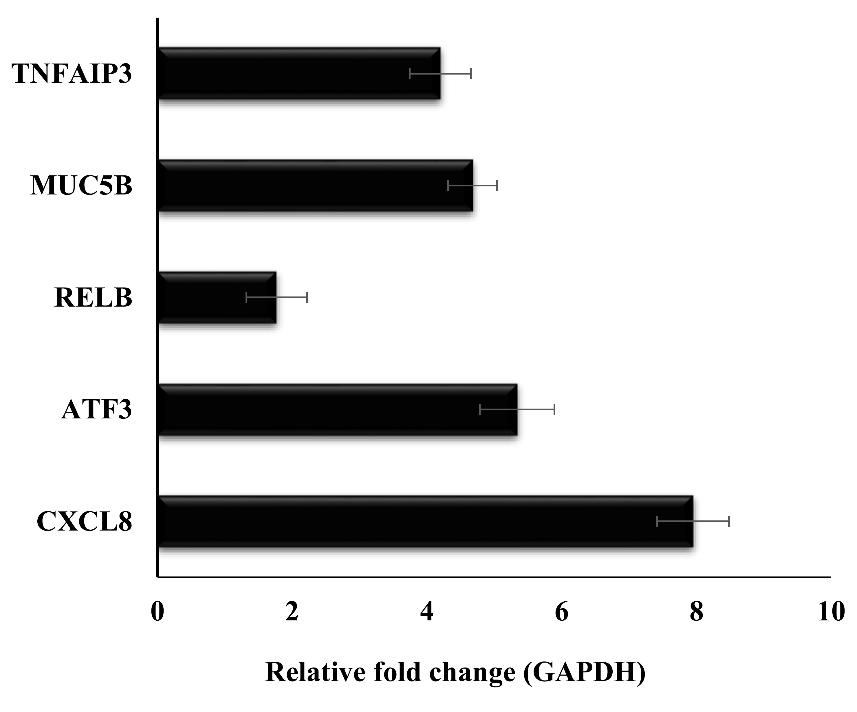

Supplement: Supplementary file 1 — Supplementary dataset 1 [file 41598_2019_41435_MOESM1_ESM.docx]
